# Spatio-temporal reconstruction of gene expression patterns in developing mice

**DOI:** 10.1242/dev.204313

**Published:** 2025-02-21

**Authors:** Laura Aviñó-Esteban, Heura Cardona-Blaya, James Sharpe

**Affiliations:** ^1^ European Molecular Biology Laboratory (EMBL-Barcelona), Barcelona 08003, Spain; ^2^ Barcelona Collaboratorium for Modelling and Predictive Biology, Barcelona 08005, Spain; ^3^ Institució Catalana de Recerca I Estudis Avançats (ICREA), Barcelona 08010, Spain

**Keywords:** Mouse development, Interpolation, Gene expression patterns

## Abstract

Understanding gene regulation in organism development is crucial in biology. Techniques like whole-mount *in situ* hybridization can reveal spatial gene expression in organs and tissues. However, capturing time-lapse movies of gene expression dynamics in embryos developing *in utero*, such as mice, remains technically challenging beyond the early stages. To address this, we present a method to integrate static snapshots of gene expression patterns across limb developmental stages, creating a continuous 2D reconstruction of gene expression patterns over time. This method interpolates small tissue regions over time to create smooth temporal trajectories of gene expression. We successfully applied it to a number of key genes in limb development, including *Sox9*, *Hand2*, and *Bmp2*. This approach enables a detailed spatio-temporal mapping of gene expression, providing insights into developmental mechanisms. By estimating gene expression patterns at previously unobserved time points, it facilitates the comparison of these patterns across samples. The reconstructed trajectories offer high-quality data that will be useful to guide computational modeling and machine learning, advancing the study of developmental biology in systems where real-time imaging is technically difficult or impossible.

## Teaser

**Highlighted Article:** A novel method for reconstructing spatio-temporal gene expression patterns in internally developing embryos, providing a tool to explore developmental processes and generate high-quality data for computational modeling.

## INTRODUCTION

The role of genes in regulating the development of a fully formed functional organism is a central subject of interest in biology. Morphogenesis can only be fully understood by characterizing the sequential unfolding of its dynamic processes, such as the regulatory interactions between genes and the patterns of gene expression over time (Jaeger et al., 2004; Balaskas et al., 2012).

**Fig. 1.**
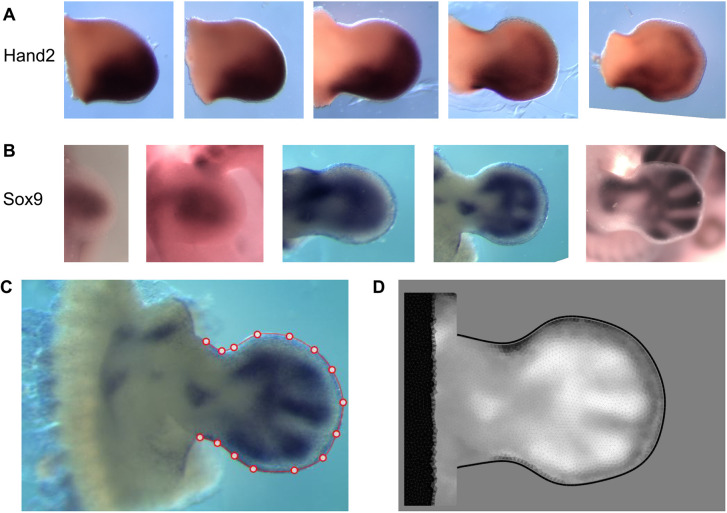
**Gene expression patterns in the limb bud.** (A) *In situ* hybridizations of *Hand2*. Morphometric stage (defined by Musy et al., 2018) from left to right: mE10:21, mE11:01, E11:10, mE11:18 and mE12:00. The gene expression pattern in present on the anterior (bottom in this view) part of the limb. (B) *In situ* hybridization of *Sox9*. Developmental stage from left to right: mE10:11, mE11:00, mE11:12, mE12:00 and mE12:12. The expression patterns of this gene converge to the skeletal pattern of the limb. (C) Staging method presented by Musy et al. (2018) and by Matyjaszkiewicz and Sharpe (2024 preprint). Red dots define the outer shape of the limb (defined by the user). (D) Digitization of the gene expression pattern on digital meshes. White represents high expression levels; black represents low expression levels.

Gene expression patterns (GEPs) provide insights into the mechanisms of development. They can be visualized by labeling the target organ (or whole embryo) using techniques, such as whole-mount *in situ* hybridization, that reveal which cells express specific genes. Genes that play crucial roles in organ development often exhibit distinctive patterns of expression. For example, in the developing limb bud, *Hand2* is initially expressed in most posterior cells and has been shown to influence the posterior state of the limb bud (Galli et al., 2010) (Fig. 1A). Similarly, *Sox9* is crucial for cell differentiation into cartilage, and its expression dynamically evolves during limb development, gradually forming a pattern that mirrors the spatial arrangement of the skeleton (Akiyama et al., 2002) (Fig. 1B).

Defining the boundaries of gene expression patterns (GEPs) presents a biological challenge. Firstly, gene expression can vary in intensity continuously across the domain. Consequently, delineating the spatial extent of gene expression can be ambiguous. Many genes in limb development exhibit blurry edges, with some showing long gradients of expression across the tissue, such as *Wnt5a* (Summerhurst et al., 2008), while others display more subtle gradients. Secondly, many gene pattern boundaries do not align with anatomical boundaries. GEPs can dynamically shift through the tissue over time as cells regulate gene expression dynamically during development (Harfe et al., 2004; Scotti et al., 2015).

Accurate modeling of such a dynamical system requires a detailed description of the process. In externally developing organisms, such as zebrafish or *Drosophila*, it is feasible to capture time-lapse movies of the entire developmental process *in vivo* in real-time (Tomer et al., 2012; Royer et al., 2016). However, for internally developing embryos, this capability is currently limited beyond the gastrulation stage (Colas and Sharpe, 2009). Moreover, *in vitro* culture techniques often fail to replicate full embryogenesis due to technical constraints. For example, in mouse, it is very difficult to mimic late embryonic stages beyond E10.5 *in vitro* (McDole et al., 2018).

One approach to address this challenge is by imaging fixed embryos, and their gene expression patterns, at different time points, gathering sufficient data to capture all dynamics. However, this approach relies on collecting a substantial amount of labeled image data, which may be time-consuming and may also not be necessary for all genes. Additionally, creating such a dataset presents its own challenges. Firstly, biological noise is a significant factor. Even embryos from the same developmental time point may exhibit slight differences due to natural variation. Secondly, the rate of developmental progression can vary between embryos, even within the same litter. The developmental age of each embryo must be determined post-harvest using methods that estimate age based on observable features (Musy et al., 2018) (Fig. 1C). Finally, a sufficient number of embryos must be harvested to ensure good coverage across different time points.

In this work, we present a computational technique that allows us to construct a smooth, continuous description of GEPs over time and space with high temporal resolution. This is achieved by integrating static data samples collected from multiple individuals through computational tracking of moving tissue segments. To validate the efficacy of this method, we have applied it to key genes that are crucial for limb development and patterning, including *Sox9*, which is a marker of skeletal progenitor cells. These reconstructed trajectories will provide a valuable reference for future modeling endeavors, significantly enhancing our ability to understand and predict developmental processes. Additionally, to demonstrate that the technique is not limited to the limb bud, we have also performed a proof-of-concept on a few dynamic genes in the developing neural tube.

## RESULTS

GEPs are spatial regions where a specific gene is expressed in the tissue. However, mapping GEPs from one time point to the next is challenging (see changing expression patterns in Fig. 1). This difficulty arises because GEPs depend on the underlying cells and the movement of these cells over time. We therefore decided to adapt an interpolation approach focusing on how small tissue regions move over time. We broke the problem into many separate ‘tissue tracks’ or trajectories, instead of dealing with the full 2D pattern at each time point. This approach is a coarse-grained version of tracking individual cell movements and their gene expression changes over time. This is a reasonable approximation since genes in the limb mesenchyme do not have sharp on-off boundaries of expression. By calculating smooth temporal trajectories for how each small tissue region regulates gene levels (on, off, or intermediate), we can recombine all tracks in their correct spatial positions over time to recreate the full 2D GEP at any point. Consequently, we have transformed the interpolation task from a spatial problem to a largely temporal one.

### Tracking tissue trajectories

**Fig. 2.**
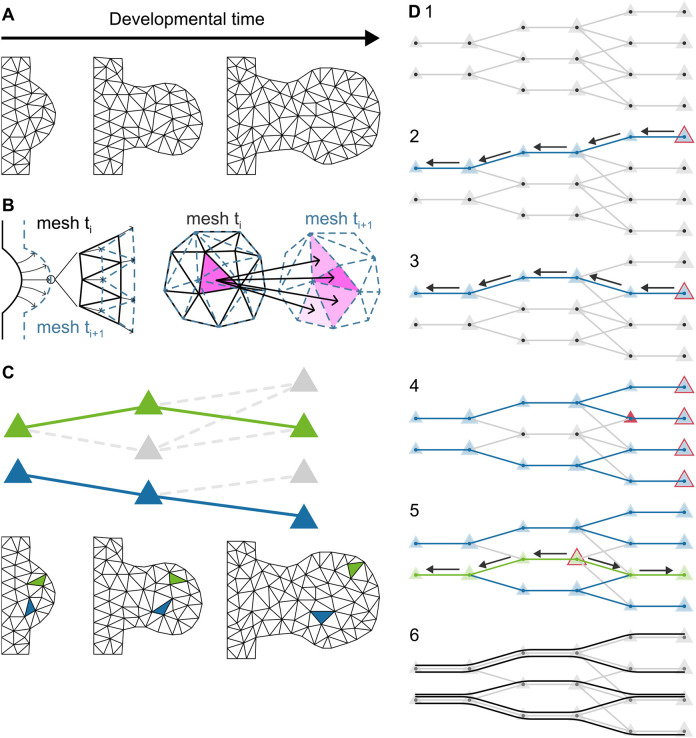
**Tissue tracks.** (A) Morphomovie toy example. The actual morphomovie has a higher resolution (i.e. each element has a smaller area). (B) (Left) Every 10 developmental minutes, the mesh moves slightly to adjust to the growth and the new outer shape of the limb. The solid black line shows the outer contour at time t and the dashed line shows the contour at time t+10 (in minutes). The triangles stretch to fill the new area. (Right) Every 60 developmental minutes, there is an extra step of remeshing to maintain constant triangle size. The solid black line shows the outer contour at time t and the dashed line shows the contour at time t+10 (in minutes). Each triangle can split into multiple triangles at the next time point, and one triangle can come from different triangles in the previous time point (adapted, with permission, from Matyjaszkiewicz and Sharpe (2024 preprint). (C) We can follow trajectories of triangles as a proxy for tissue movement in space. (Top) Representation of the morphomovie as a Directed Acyclic Graph (DAG), showing predecessors and descendants of each triangle. (The two colors indicate two different example trajectories.) (Bottom) Spatial distribution of the trajectories. (D) Representation of the algorithm to derive the trajectories from the morphomovie. The size of the triangle illustrates growth. (1) Representation of the Morphomovie as a DAG. (2) One triangle at the last time point is picked and its trajectory tracked backwards by selecting the predecessors across developmental time. (3) This is repeated for the next triangle at the last time point. If one triangle has multiple predecessors (e.g. the red triangle), the algorithm selects the one with the higher area overlap as the predecessor. (4) This is repeated for all triangles at the last time point. (5) Some triangles at intermediate time points are not picked in this process. For each of them, a similar process as above is repeated, but this time both backwards and forwards, so that all triangles are included in at least one trajectory, and all trajectories are complete from the first time point to the last. (6) The different trajectories found in this toy example.

To represent the growing tissue, we used a collection of meshes called a morphomovie, a technique previously published by Marcon et al. (2011) and explained in the Materials and Methods. From the morphomovie, we extracted 42,155 ‘tissue tracks’ over time. The triangles of each mesh represent both a small unit of space (a sub-region of the limb at that time point) and also a unit of time (as each triangle only exists for 1 h until the limb tissue is re-meshed) (Fig. 2). A tissue track is therefore a sequence of triangles in chronological order, each successive triangle being an approximation of how a particular piece of tissue moves over time. Choosing which triangles should be linked together into useful trajectories involves an algorithm that calculates the maximum overlap between triangles of adjacent time-points. Tissue tracks are not entirely independent – a triangle may be included in more than one trajectory. This can largely be explained by the fact that the tissue grows over time, and therefore the last mesh has many more triangles than the first mesh. This is a simple reflection of the fact that the limb bud has many more cells at the end than at the beginning. Multiple trajectories will therefore share the same triangles at early time points. This effect may also happen at later time points, as our mesh discretization of space is coarse grained. But this is not a problem, since our goal is to find average tissue movements, not to calculate long-term fate maps.

### Intensity mapping and interpolation

After obtaining the many individual trajectories of small tissue regions (the different triangles of the meshes over time), we fitted the gene intensity data. We started with *Sox9*, a skeletal marker of the limb that shows high spatio-temporal complexity. Our method can use images from different types of gene expression analysis. In this case, we used mostly classical BCIP/NBT whole-mount *in situ* hybridization (Luis de la Pompa et al., 1997), but also a number of fluorescence 3D *in situ* hybridization chain reaction (HCR) datasets (Morabito et al., 2023). These latter images were created by calculating 2D projections of the HCR volumes (Fig. S1). We used the staging system from Musy et al. (2018) to semi-automatically assign a developmental age to each image. Then, to align the limb bud to the morphomovie, we morphed each image to the mesh corresponding to the assigned stage using the methods of Matyjaszkiewicz and Sharpe (2024 preprint). This step estimates relative gene expression levels for all triangles at that time point. It is important to note that the experimental image data do not have uniform intensity levels across time. Each time point has a variable number of images, and some have none. This highlights the value of smooth interpolation over time. After the prepossessing of the images, the data included 204 digitized images.

**Fig. 3.**
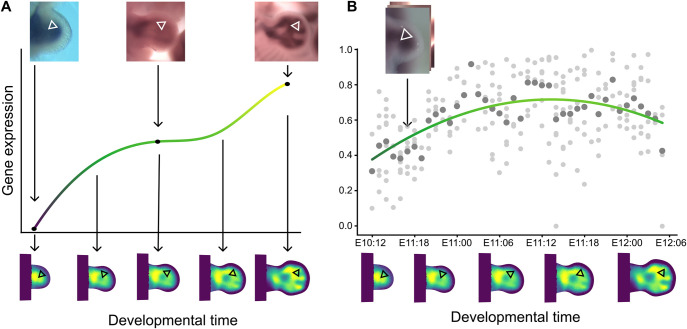
**Gene expression interpolation.** (A) Schematic of the process. (Top) Raw data for *Sox9 in situ* hybridization. White triangles represent a tissue track. (Middle) Gene expression levels are obtained from the data of triangles in the tissue track, then interpolated, represented by the line. (Bottom) The interpolation is mapped back to the morphomovie. (B) Real trajectory example. Lighter dots show the raw data. Darker dots show the mean value for each time point. The line shows the B-Spline interpolation.

For each trajectory, we needed to smooth and infer the missing gene expression data using interpolation methods. A polynomial fit, which gives equal weight to all points, could be used, but it would struggle to capture subtle changes due to the constraints of the selected polynomial degree. Moreover, there is no prior polynomial (or curve) that the data should fit. As an alternative, piecewise linear interpolation could be used. To perform such interpolation, the mean intensity for each time point should be computed and then interpolated. This approach gives equal weight to each time point but not to each data point, and the resulting fit would not have any prior constraint. However, the result would not be continuous in the derivative, meaning a discontinuous rate of gene expression change, which we consider not biologically meaningful. Therefore, we chose to use B-splines, an interpolation technique that uses four neighboring points to define the sections, ensuring the final curve is continuous in the derivative by definition. We used Vedo (Musy et al., 2024) to perform the interpolation. Fig. 3A shows a graphical representation of the interpolation step. In Fig. 3B, a real example trajectory is shown.

**Fig. 4.**
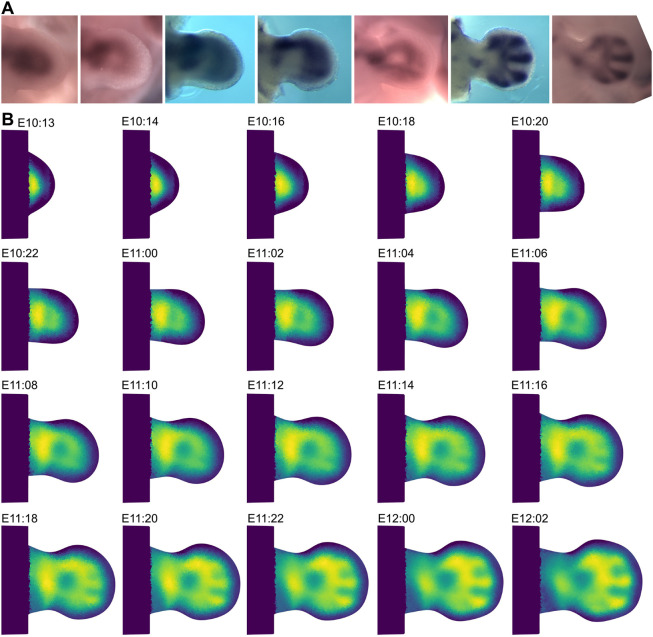
***Sox9* reconstruction.** (A) Subset of the *Sox9* raw dataset. (B) Reconstruction of this gene from stage mE10:13 to mE12:02. Each reconstruction frame represents two developmental hours apart from the first and second images, which represent one developmental hour lapse.

Once each trajectory was interpolated, the values were mapped back onto the standard morphomovie. Triangles that were part of more than one trajectory are averaged. This provided a full 2D and time-continuous reconstruction of the *Sox9* gene (Fig. 4).

### Refine the whole pattern using intensity optimization (boot-strapping)

**Fig. 5.**
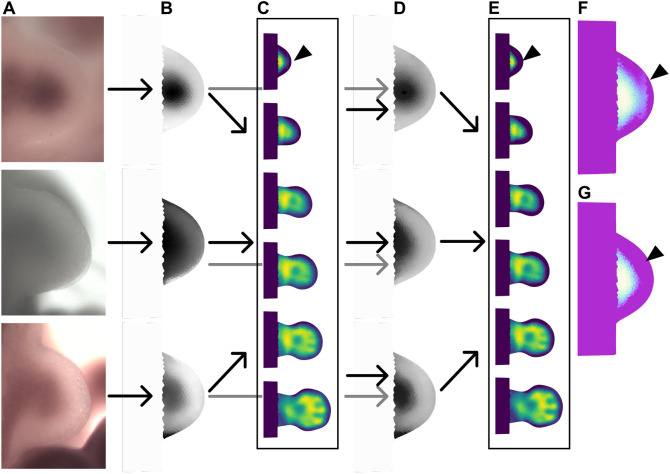
**Refinement process.** (A) Raw data. Three images of Sox9 at the same time point, mE10:14, for which the variation in intensities is evident. (B) Digitization of the raw data. White represents no expression and black represents high expression. The shape of the expression pattern is similar, yet the intensities show high variability. (C) Initial reconstruction using the digitization of the raw data. (D) Matching of the intensities of the raw data to the intensity values of the reconstruction at the same time point (mE10:14) shows highly improved digitization compared to B. (E) Final reconstruction using the boot-strapped data. The process improves the quality of the reconstruction, e.g. correcting the artifact indicated by the arrowhead. (F) Zoomed image of the interpolation at stage mE10:12 before the bootstrapping. Owing to the nature of the raw data and the semi-automated processing, there is an artifact in the noise (arrowhead). (G) Zoomed image of the interpolation at stage mE10:12 after the bootstrapping. After the bootstrap step, the artifact in the noise at mE10:12 disappears (arrowhead).

One challenge to address is that the raw data have inconsistent intensity levels from one image to the next due to experimental limitations and variation. In theory, this should not be a major concern, as the information content we can obtain from *in situ* hybridization resides in the shape of the GEPs rather than their absolute expression levels. Nevertheless, in practice it could reduce the quality of the result, and we therefore explored a boot-strapping approach to reduce this problem. To polish the overall pattern, we performed one cycle of bootstrapping, a refinement pass of the raw digitized data to reduce initial variation in expression intensity. Given that we cannot know an ‘absolute correct’ set of expression levels for the data, it is therefore not obvious how to normalize the raw images *per se*. However, after estimating a first version of the GEP trajectory (previous section, and Fig. 4), this trajectory could be used as a template on which to normalize all the original raw images. Afterwards, a new improved version of the trajectory from the new set of adjusted images can be produced. To perform normalization, we used histogram matching of the intensities of each raw image with the same time point from the first version of the trajectory. Once all raw images had been adjusted, we used this new set to create a new reconstruction. Fig. 5 graphically shows this procedure, and the initial and final reconstructions for *Sox9* (Movie 1). As displayed in Fig. 5, we managed to reduce initial noise, and also to remove some artifacts on the initial reconstruction.

### Application of the method to other genes

**Fig. 6.**
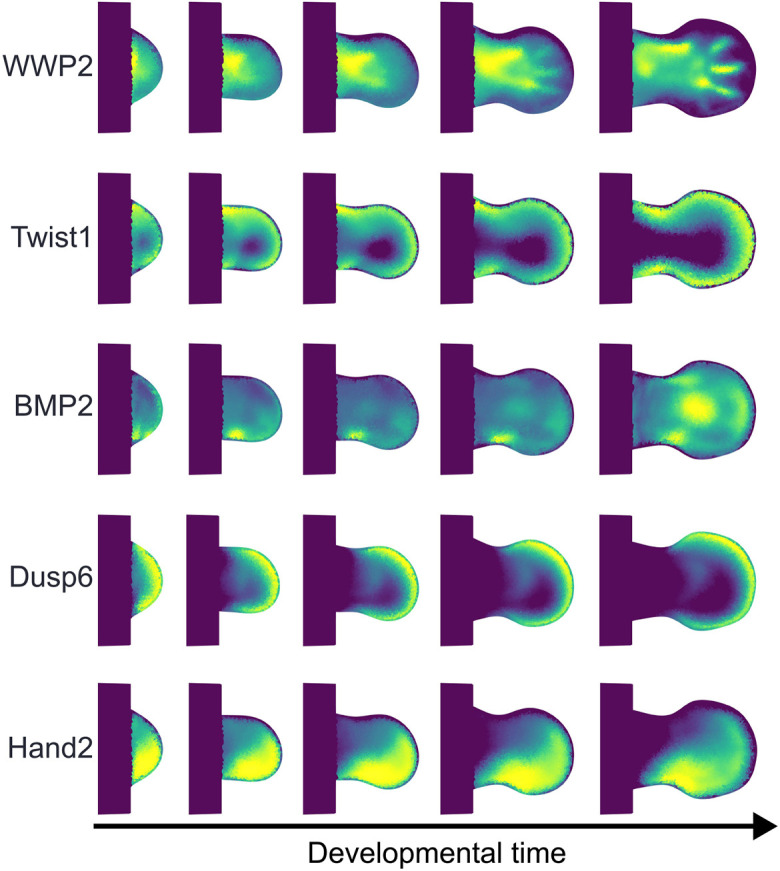
**Application to other genes.** From left to right, the developmental stages are mE10:13, mE10:22, mE11:07, mE11:16, and mE12:02. The method is able to capture simple as well as complex patterns. Movies 2-6 show, respectively, the full reconstruction.

To showcase the generality of the approach, we generated 2D reconstructions of key developmental genes for the limb, including the anterior-posterior axis marker *Hand2* (Osterwalder et al., 2014), a limb bud mesenchyme regulator *Twist1* (Wade et al., 2012), two chondrogenesis formation regulators, *Bmp2* (Bénazet et al., 2012; Lopez-Rios et al., 2012) and *Wwp2* (Mokuda et al., 2019; Nakamura et al., 2011), and a FGF downstream target, *Dusp6* (Kawakami et al., 2003; Li et al., 2007). We staged and digitized the images for these new genes. Then, we automatically applied the same algorithm that we used for *Sox9* to produce the reconstructions of each of these genes. As shown in Fig. 6, we successfully managed to generate a smooth reconstruction for each of these genes, without any manual parameter tuning of the algorithm. Movies 2-6 show the reconstruction of these gene expression patterns for *Wwp2*, *Twist1*, *Bmp2*, *Dusp6*, and *Hand2*, respectively.

### Relationship between complexity of pattern and amount of image data required

An interesting question that arises from this approach is to asses or quantify how many images are needed to create a reasonable trajectory for a given gene. As seen in the previous sections, each gene presents different spatial patterns over time. Some have complex shapes, while others have simpler ones. It is therefore expected that the amount of data required to reconstruct the trajectory will vary: a simple pattern may not require many images, while a complex pattern will require a larger amount of data.

**Fig. 7.**
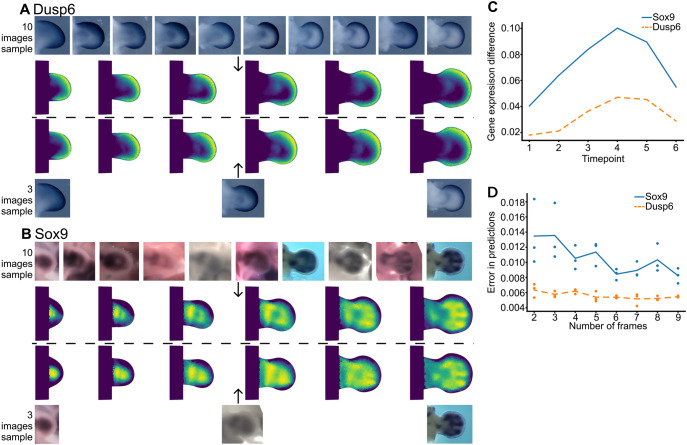
**Reconstruction with different numbers of images.** (A) *Dusp6* reconstruction. (Bottom) Three selected raw images and their reconstruction. (Top) Ten selected images and their reconstruction. (B) *Sox9* reconstruction. (Bottom) Three selected images and their reconstruction. (Top) Ten selected images and their reconstruction. (C) Comparing reconstructions of the same gene. We computed the residuals at each time point for reconstructions of the same gene. Solid line indicates *Sox9* residuals; dashed line indicates *Dusp6* residuals. (D) The *x*-axis shows the number of randomly picked frames used in the reconstructions. Each dot represents the residuals between five randomly selected images and the reconstruction.

We explored this by picking two genes: *Dusp6* (simple pattern) and *Sox9* (complex pattern). We selected 10 images for each gene, evenly distributed over time and applied our interpolation algorithm. Six time points of the interpolated trajectories are shown in Fig. 7A (Dusp6) and Fig. 7B (Sox9). Next, we chose a subset of only 3 of the raw images (the same initial and last images, and one in the middle of the sequence) and repeated the process. Fig. 7 shows that, with 10 images, we could reproduce the spatial dynamics for both *Sox9* and *Dusp6*. When reducing to only 3 input images, the trajectory of *Dusp6* was almost identical to the 10-image reconstruction. However, reducing *Sox9* to three images significantly impaired reconstruction quality, especially in the middle of the sequence. To go beyond visual inspection, we performed a quantitative comparison of the two reconstructions for each gene. The difference in pattern was calculated at each of the six time points shown in Fig. 7, by summing the differences at every triangle in the mesh. Confirming what can be seen by eye, the difference in Sox9 pattern between the 10-image interpolation and the 3-image interpolation is greatest for the medium time points (Fig. 7C). In other words, when only 3 input images were used, the resulting interpolation diverges significantly from the result obtained when using 10 images. The comparison for Dusp6 also shows an increase for medium time points, but the overall differences are much lower. Since Dusp6 is a much simpler pattern, fewer data points can nevertheless provide a good result. This suggests that, in general, complex patterns need more empirical data for accurate reconstruction.

To understand how the number of images affects the final reconstruction, we carried out a more comprehensive analysis – going beyond comparing only two amounts of input data (10 input images versus 3), and instead performing comparisons for the full range of the amounts of input data – from 4 input images to 10. We always selected the youngest and oldest image for each gene. To ensure each subset contained at least 4 unique time points, which is required for B-spline interpolation, we started with two intermediate random frames. For each subset, we compiled relevant data and performed a reconstruction. To calculate difference values, we needed to compare each predicted trajectory to some control trajectories. These control trajectories were calculated by randomly selecting five images from the original full dataset, excluding those used in the reconstruction. To reduce the difference due to intensity profile variation of the raw data, we minimized the intensity difference between the reference and raw data by adjusting the minimum and maximum intensity values of the raw data. As shown in Fig. 7D, *Dusp6* exhibits a flatter error line, indicating that its reconstruction is relatively stable regardless of the number of images used. In contrast, *Sox9* shows a significant decrease in errors as more data are added, highlighting the need of increased data for improving reconstruction accuracy for complex gene patterns. Additionally, for each particular number of frames used, *Sox9* displays higher variability in error measurements compared to *Dusp6*. This suggests that the reconstruction of complex shapes, as represented by *Sox9*, is always less robust than that of simpler shapes such as those of *Dusp6*.

## DISCUSSION

A fascinating feature of gene expression patterns is that they do not necessarily correspond to fixed anatomical boundaries and can dynamically move through tissue during development. A specific cell might switch a gene on and later switch it off again as the pattern ‘shifts’ through that region of the tissue like a wave. In other words, the movements of tissue and GEPs do not always correlate.

In this study, we present a pipeline to reconstruct biologically meaningful continuous 2D and time-spatiotemporal GEPs from discrete snapshots of expression in the developing mouse limb. The results provide valuable insight into the temporal development of each gene. Continuous descriptions of gene expression offer a simple visualization of complex data, making it easier to manage and interpret while preserving overall gene activity dynamics that may be missed with discrete measurements. This technique enhances understanding of the complex, gradual changes in gene expression over time and space, enabling researchers to observe shifts at different stages and to pinpoint critical transitions and thresholds.

Moreover, this tool facilitates the simultaneous analysis of multiple genes, allowing for comparisons of correlations and spatial patterns. Ideally, these comparisons should be made at the same time point. Our technique enables the estimation of a GEP at time points for which raw data may be unavailable. As a result, it produces a more comprehensive, holistic view of the biological process. Additionally, this method can improve computer modeling of the system. Comparing multiple genes provides researchers with more information to generate hypotheses about their spatio-temporal relationships. The final reconstructed trajectories can also guide data-driven computational modeling and machine learning, as we have previously demonstrated with low-resolution reconstructions (Uzkudun et al., 2015).

Additionally, another potential application of this method is in interpolating gene expression patterns in mutant samples, which could enhance the description of these patterns even with limited data points. However, this approach is most effective when the overall morphology of the sample remains largely unchanged, as it relies on interpolation within the wild-type morphomovie. If significant morphological differences are present, the method may not provide accurate representations of the gene expression patterns, highlighting the need for careful consideration of morphological variations in such analyses (Martínez-Abadías et al., 2015).

Another key aspect of our study is the variety of input data available. The interpolation algorithm presented in this paper is agnostic to data types, as long as they can be mapped to the correct space and time of the system. Depending on how the final reconstruction is used, the user should decide which data type to interpolate. In this study, we primarily use 2D whole-mount *in situ* hybridization (WISH) images due to the large availability of this type of data, and we complement these with a few state-of-the-art HCRs for *Sox9*, as our main goal was to reconstruct the overall pattern shape. However, we could integrate other data types, such as quantitative transcriptomics, to better quantify gene expression over time or to use spatial transcriptomics data. Nevertheless, it is important to note that the primary purpose of this technique is to interpolate data across multiple timepoints, so other data types (such as spatial transcriptomics) will only be useful if captured at multiple different developmental time points. Additionally, multi-channel HCRs could provide better relative gene expression levels across different genes.

We have also provided a concrete demonstration of an intuitive idea – that the number of images needed for a good interpolation depends on the complexity of the GEP. Even though the number of images needed for a particular gene cannot be predicted beforehand, patterns that are highly dynamic or spatially complex will need more images.

We believe this approach could be valuable for any model system where capturing time-lapse movies of gene expression dynamics is unfeasible, and static images are the primary data source. This includes any project for which live transgenic reporter constructs cannot be used, or any developmental system that does not develop normally in culture, e.g. all later-stage mammalian embryos. To demonstrate that our technique is not limited to limb development, we also performed a proof-of-concept analysis (Yokoyama et al., 2009) of a few genes expressed during the development of the mouse neural tube (see Movies 7-10 and Fig. S2). The technique will therefore be useful for the study of many species – helping to fill in interpolated data from time points that have not been captured. The main limitation of the method lies in the necessity of an independent staging, aligning and digitizing the original data to a standard reference. However, the tools provided here suggest that this method could indeed be applied to other model systems. In principle, the same approach could also be generalized to 3D data by tracking tissue movements and interpolating intensity values for each track, providing that suitable 3D data exist.

## MATERIALS AND METHODS

### Materials

In this study, we utilized a comprehensive dataset obtained from the Sharpe lab database, which includes *in situ* hybridization (Luis de la Pompa et al., 1997) and *in situ* HCR (Morabito et al., 2023) data collected over different projects. This dataset includes a wide range of gene expression patterns at various developmental stages for both mouse hindlimb and forelimb. The data were manually curated to ensure accuracy and consistency.

### Animal studies and ethics

Mouse embryos were needed to perform *in situ* HCR. C57BL/6J were bred and C57BL/6J female mice were humanely euthanized at various gestational time points from E10.5 to E12.5 to collect embryos. The embryos were dissected and immediately fixed overnight at 4°C in 4% PFA on a shaker. After fixation, the embryos were washed twice with 0.1% PBT and gradually dehydrated using a series of methanol dilutions in PBT (25%, 50%, 75%, and 100%) before being stored at −20°C. All procedures, including euthanasia and embryo collection, were conducted in accordance with the guidelines set by European and institutional ethical committees. All animal procedures presented in this study were approved by the Committee for Animal Welfare and Institutional Animal Care and Use (IACUC) at EMBL.

### Methods

#### HCR sample preparation

Samples were cleared using a fructose-glycerol solution and subsequently embedded in 1% agarose prepared in 10 mM Tris buffer at pH 7.4. The embedded samples were trimmed into octagonal prisms of ∼1 cm^3^. These prisms were then placed in a 24-well plate containing fructose-glycerol solution to dissolve the agarose, a process carried out for a minimum of 24 h at room temperature protected from light.

#### HCR data acquisition

Imaging was conducted using a MuViSPIM light-sheet microscope (Luxendo). Dual-side illumination was performed with the illumination objective Nikon CFI Plan Apo Lambda 4× and the collar was adjusted to 1.47 to match the refractive index of the fructose-glycerol imaging medium. Fluorescence was captured using a 10× immersion objective Nikon CFI Plan Apo 10XC Glyc (RI adjustment range 1.33-1.51, 0.5 NA, working distance 5.5 mm). The agarose block containing the sample was mounted on a custom-made 3D-printed holder and immersed in a cuvette filled with the fructose-glycerol clearing solution for imaging.

#### The morphomovie

We previously named a sequence of meshes that represent a pre-defined growing 2D domain as a morphomovie (Fig. 2A). This was first described by Marcon et al. (2011), and subsequently used by Raspopovic et al. (2014), Uzkudun et al. (2015), and Matyjaszkiewicz and Sharpe (2024 preprint). The morphomovie used in the current study describes the mouse hindlimb bud with a set of 48 meshes, corresponding to 48 h of developmental time. Every 10 min, the vertices of the mesh are displaced, to capture the changes in limb morphology. After each 1 h interval, the 2D domain is re-meshed to maintain a consistent element size and total tissue growth as described previously (Marcon et al., 2011) (Fig. 2B). The internal tissue movements represented within the morphomovie represent normal limb development, as they were derived from real experimental data on labeled clones (Marcon et al., 2011).

#### Trajectory tracking

In our new interpolation technique described here, we focus on time more than space. Instead of working with the full 2D space at each timepoint, we track one spatial element from each time point (i.e. one triangle) over time – i.e. finding a sequence of triangles across the different time points that can represent the same piece of tissue. To achieve this, we used the following approach. Due to the growth of the tissue, the final mesh has more triangles than the initial mesh. To ensure all triangles were captured in at least one trajectory, we tracked backwards from the last time point to find the precursor triangle on the previous time point mesh. For the time points that were re-meshed, we picked the triangle with the highest area overlap. We performed this through all the time points for each of the triangles at the final time point (as shown in Fig. 2C). It is important to state, however, that because of re-meshing, some triangles at intermediate timepoints were not captured in any of this first set of trajectories. To correct this problem, after each iteration of trajectory creation, we searched for triangles not yet captured and applied the same approach to them. However, since these triangles are not in the first or last time point, we completed the ‘tissue track’ backwards and forwards, so that all the trajectories were present at all time points (Fig. 2C).

#### Software used

In this study, we used the staging system (Musy et al., 2018) and digitization through the web app LimbNET from the Sharpe Lab (Matyjaszkiewicz and Sharpe, 2024 preprint). We used Vedo (Musy et al., 2024) to perform the B-Spline interpolation, to visualize the meshes and to create the neural tube morphomovie. Graph plots were created using Matplotlib.

#### B-spline interpolation

Basis splines or B-splines, are a type of curve extensively used in computer graphics, animation, and computer-aided design. They create smooth interpolation curves by utilizing control points and specific basis functions, which manage the influence each point has on the curve. B-splines are designed to provide local control, meaning only nearby points affect the curve, unlike polynomial fits that can alter the entire curve with a single point adjustment. B-splines ensure smoothness of the curve and its derivatives (de Boor, 1980). This allow us to interpolate gene expression without abrupt changes in the expression or their rate of change.

#### Histogram matching

Given source histogram *h*_*s*_ and reference histogram *h*_*r*_, the histogram matching process involves the following steps:

(1) Compute the cumulative distribution function (CDF) of the source histogram *h*_*s*_ using

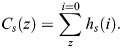
(2) Compute the cumulative distribution function (CDF) of the reference histogram *h*_*r*_ using

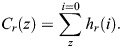
(3) Create a mapping function, *M*, such that for each pixel value, *z*, in the source image, the corresponding value *z*^′^ in the reference image is found using

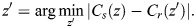


#### Reconstruction accuracy

To evaluate the accuracy of the reconstructed data, we compare it to the reference data by minimizing the sum of squared differences (SSD) between the reconstructed intensities *R*_*i*_ and the transformed reference intensities *g* (*D*_*i*_, *x*, *y*, *D*_*min*_, *D*_*max*_). The transformation function *g* is designed to adjust the intensity values of the reference data to match the range of the reconstructed data. The objective function is defined as:

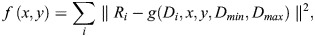
where *R*_*i*_ and *D*_*i*_ represent the reconstructed and reference intensities at mesh element *i*, respectively.

The function *g* (*D*_*i*_, *x*, *y*, *D*_*min*_, *D*_*max*_) applies a piecewise linear transformation to the reference intensity *D*_*i*_, ensuring consistency across different intensity ranges. Specifically, the transformation is defined as:

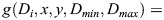


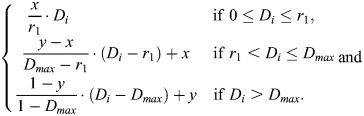


The parameters *x* and *y* are optimized using the MIGRAD algorithm, which minimizes the objective function *f*(*x*, *y*) over the constrained range [0, 1]. The optimization process iteratively adjusts *x* and *y* to minimize the SSD, thereby providing the best alignment between the reconstructed and reference data. Overall, this method allows an accurate and robust evaluation of the reconstruction quality under varying input intensity profiles.

#### Use of artificial intelligence tools

We utilized ChatGPT to assist in the drafting and refinement of the manuscript.

## Supplementary Material



10.1242/develop.204313_sup1 Supplementary information
