# Supplementary material for: Spatio-temporal reconstruction of gene expression patterns in developing mice
Source: Development. 2025 Feb 21;152(4):DEV204313. doi: 10.1242/dev.204313 (PMC11883288; doi:10.1242/dev.204313)
Supplement: Supplementary information [file develop-152-204313-s1.pdf]

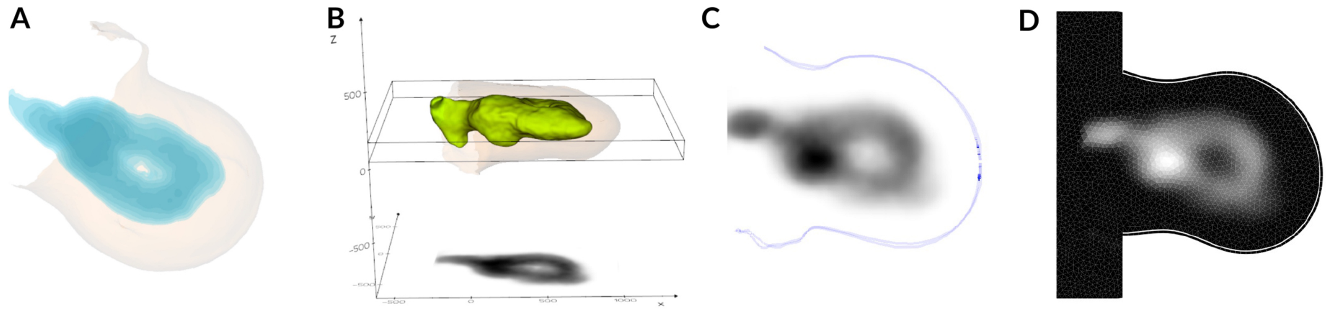

Fig. S1. Pipeline to clean HCR volumes. (A) The surface of the limb is segmented using the DAPI channel. For the Sox9 channel, the top and bottom intensity values are excluded to mitigate noise. (B) A custom tool is employed to dynamically compute the mean projection of the Sox9 expression slab.(C) The resulting projection of the slab. (D) The slab is digitized and mapped onto the morphomovie.

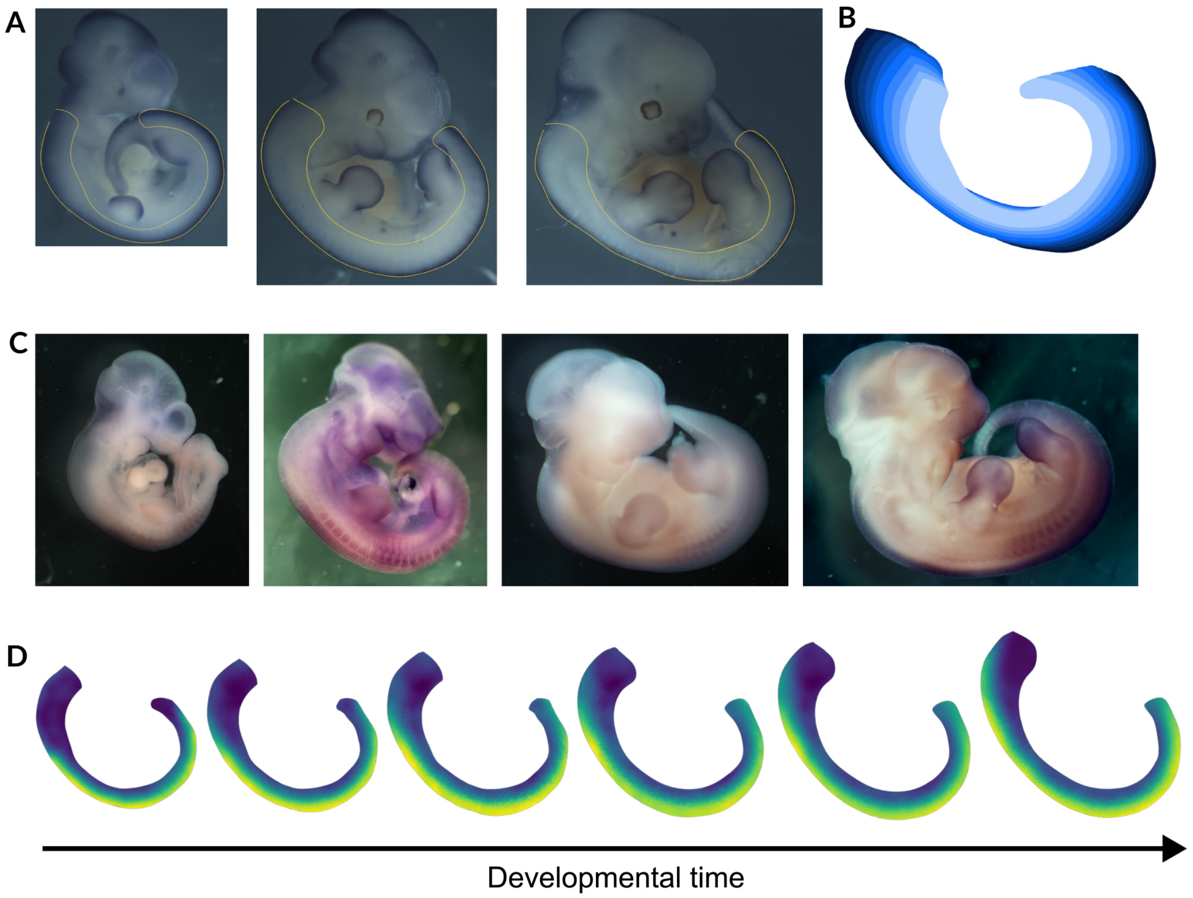

Fig. S2. Hoxa7 neural tube. (A) Selected shapes used to construct the neural tube morphomovie. (B) Interpolated shapes representing the neural tube morphomovie. (C) Hoxa7 expression data obtained from EMBRYS (Yokoyama, 2009). (D) Interpolation of Hoxa? gene expression mapped onto the neural tube morphomovie.

Development • Supplementary information

A

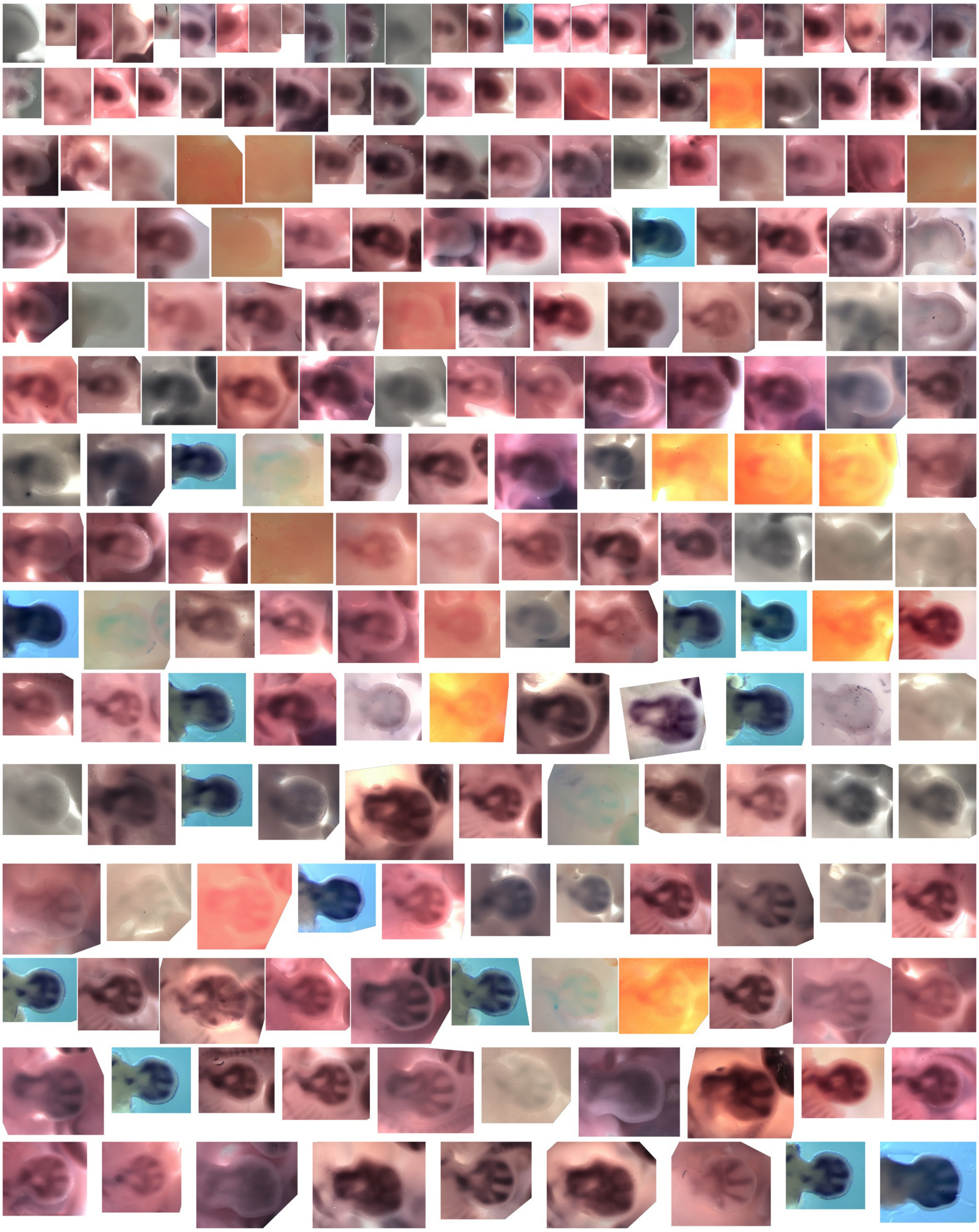

Development • Supplementary information

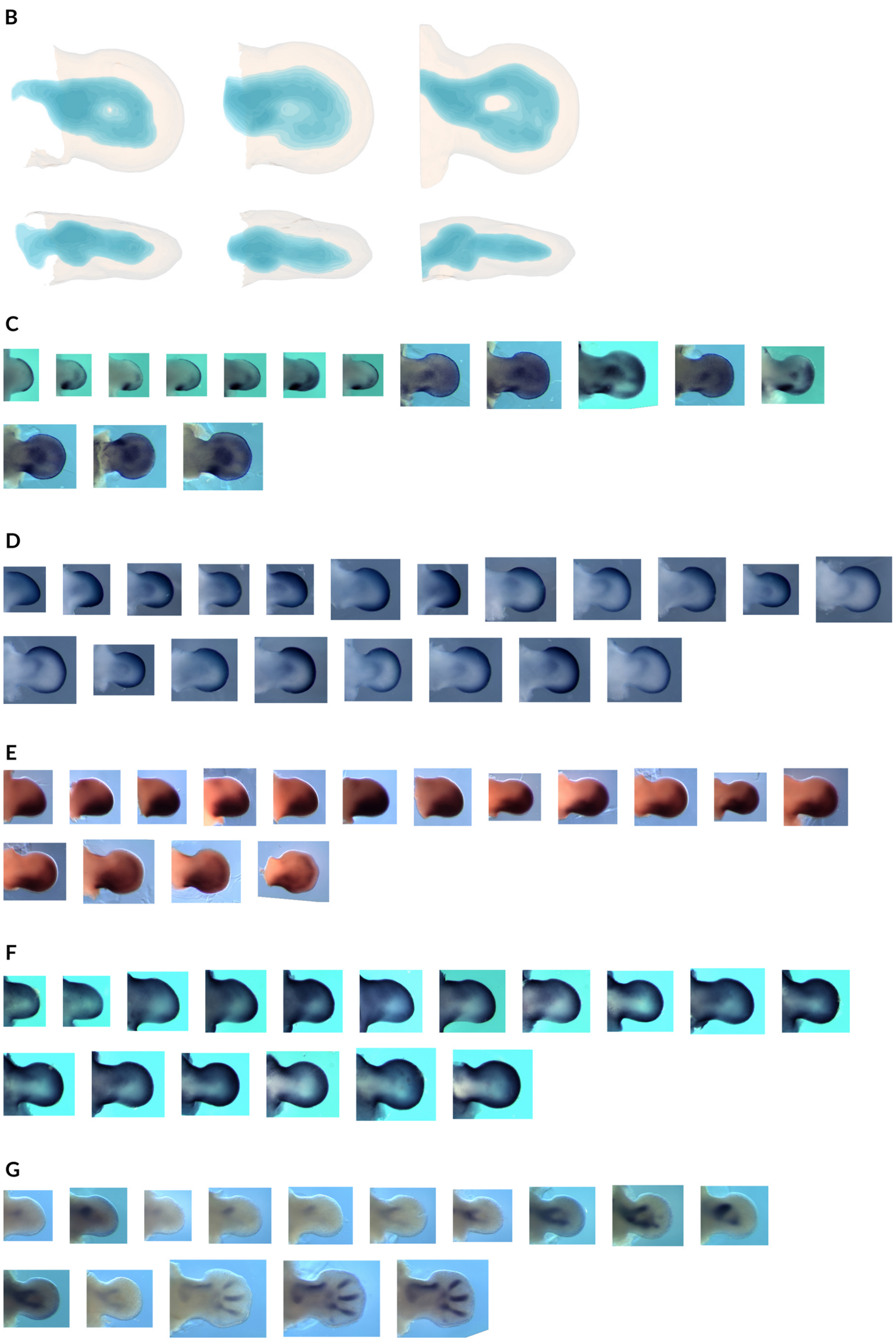

Fig. S3. Collection of images used for the Limb interpolation.

(A) Sox9 raw data collection.

(B) Sox9 clean HCR data volumes.

(C) *BMP2* raw data collection.

(D) *Dusp6* raw data collection.

(E) *Hand2* raw data collection.

(F) *Twist1* raw data collection.

(G) *WWP2* raw data collection.

Development • Supplementary information

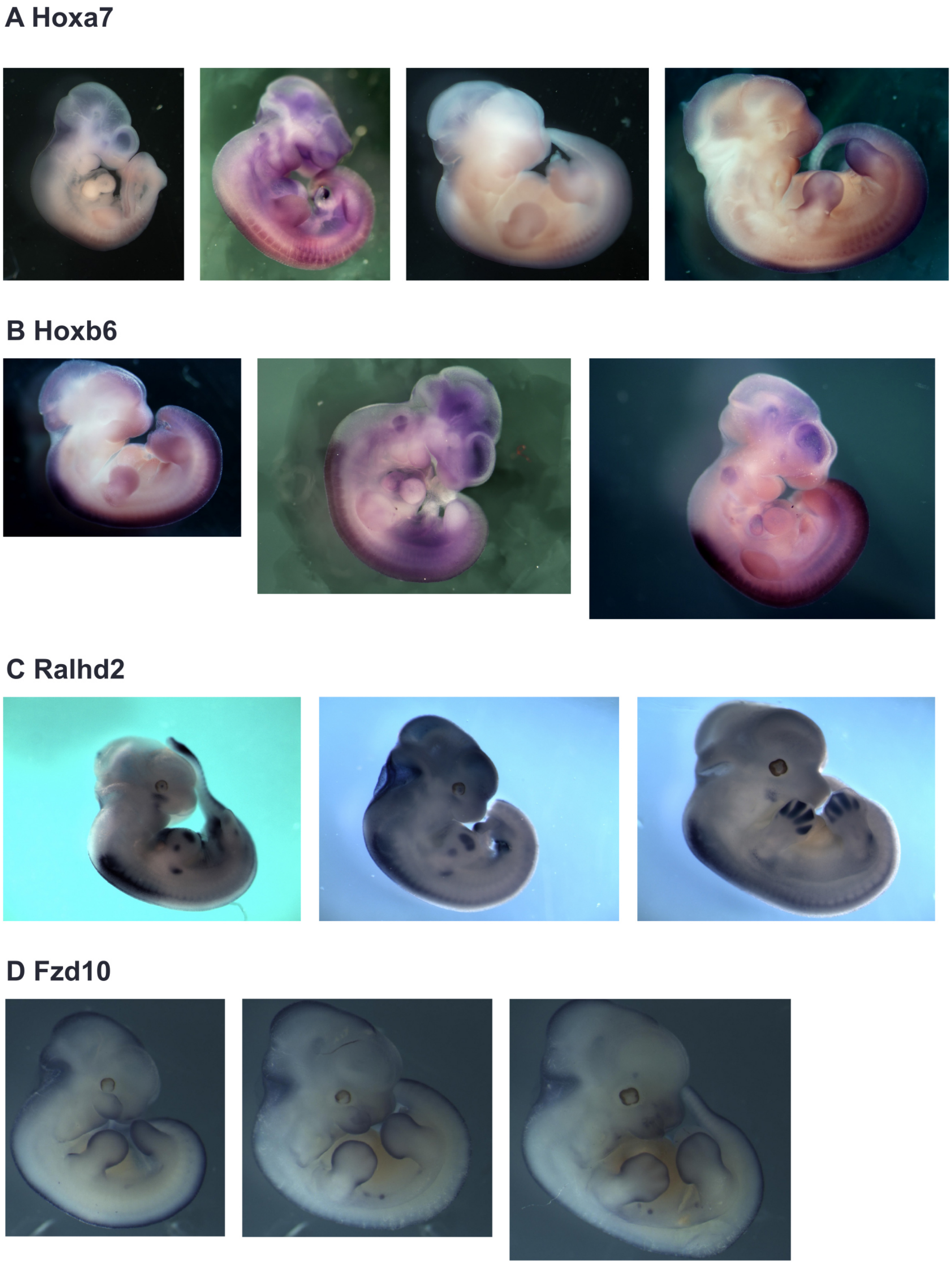

Fig. S4. Collection of images used for the Neural Tube interpolation. (A) *Hoxa7* raw data collection. Data from EMBRYS (Yokoyama, 2009). (B) *Hoxb6* raw data collection (data from EMBRYS (Yokoyama, 2009). (C) *Raldh2* raw data collection. (D) *Fzd10* raw data collection.

Development • Supplementary information

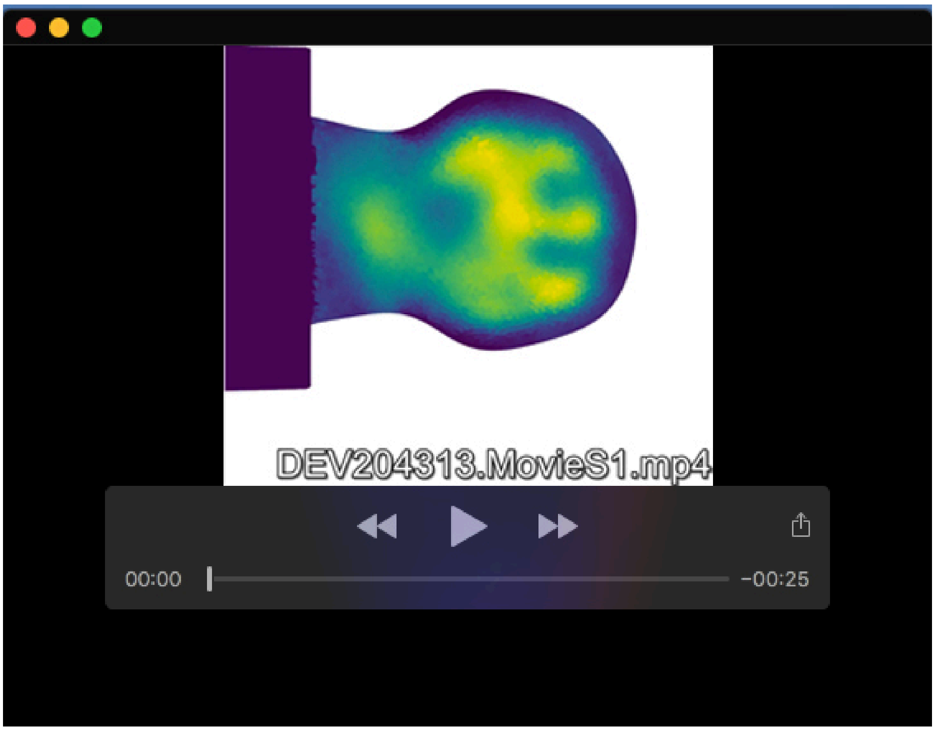

Movie 1. Interpolation of gene Sox9 expression from developmental stage E10:13 to developmental stage E12:05. The video demonstrates the temporal progression with each frame spaced one developmental hour apart. A total of 214 digitized images were utilized to create this interpolation.

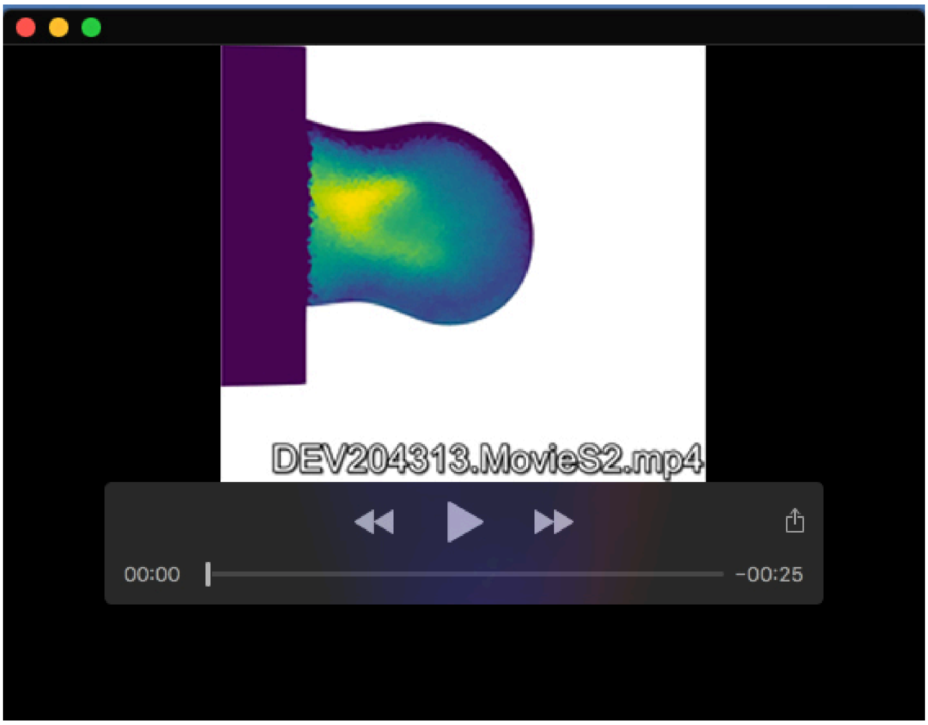

Movie 2. Interpolation of gene WWP2 expression from developmental stage E10:21 to developmental stage E12:08. The video demonstrates the temporal progression with each frame spaced one developmental hour apart. A total of 15 digitized images were utilized to create this interpolation.

Development • Supplementary information

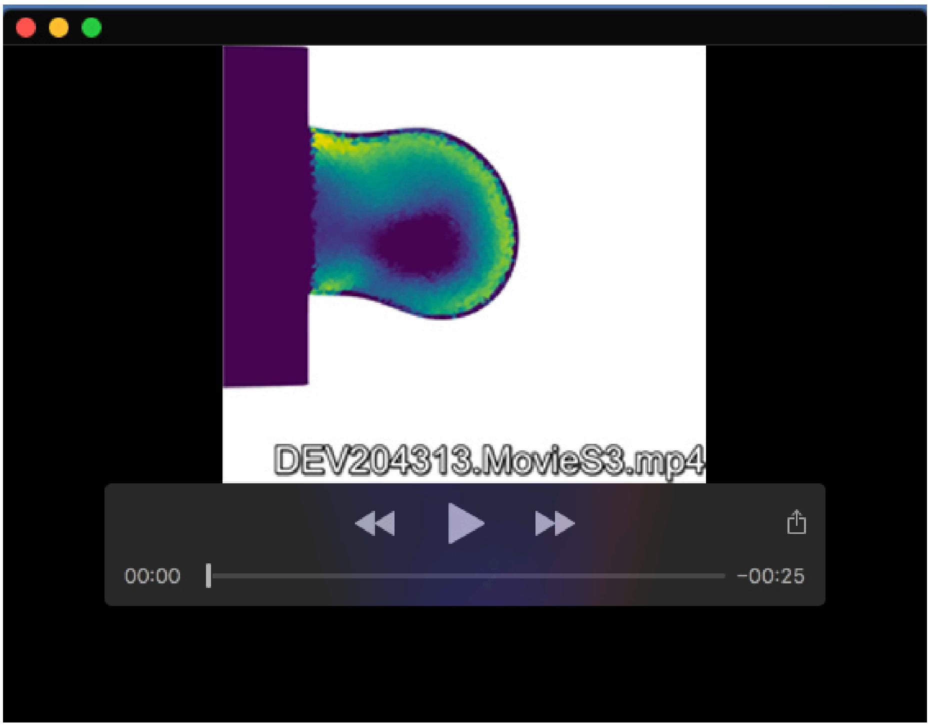

Movie 3. Interpolation of gene Twist1 expression from developmental stage E10:17 to developmental stage E11:22. The video demonstrates the temporal progression with each frame spaced one developmental hour apart. A total of 17 digitized images were utilized to create this interpolation.

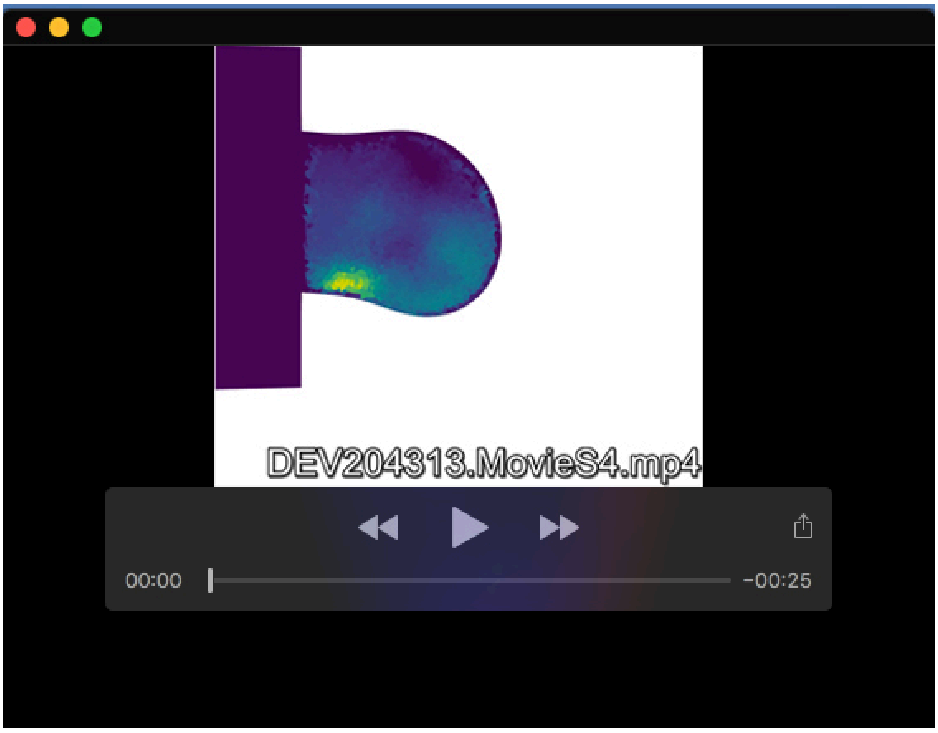

Movie 4. Interpolation of gene BMP2 expression from developmental stage E10:14 to developmental stage E12:04. The video demonstrates the temporal progression with each frame spaced one developmental hour apart. A total of 16 digitized images were utilized to create this interpolation.

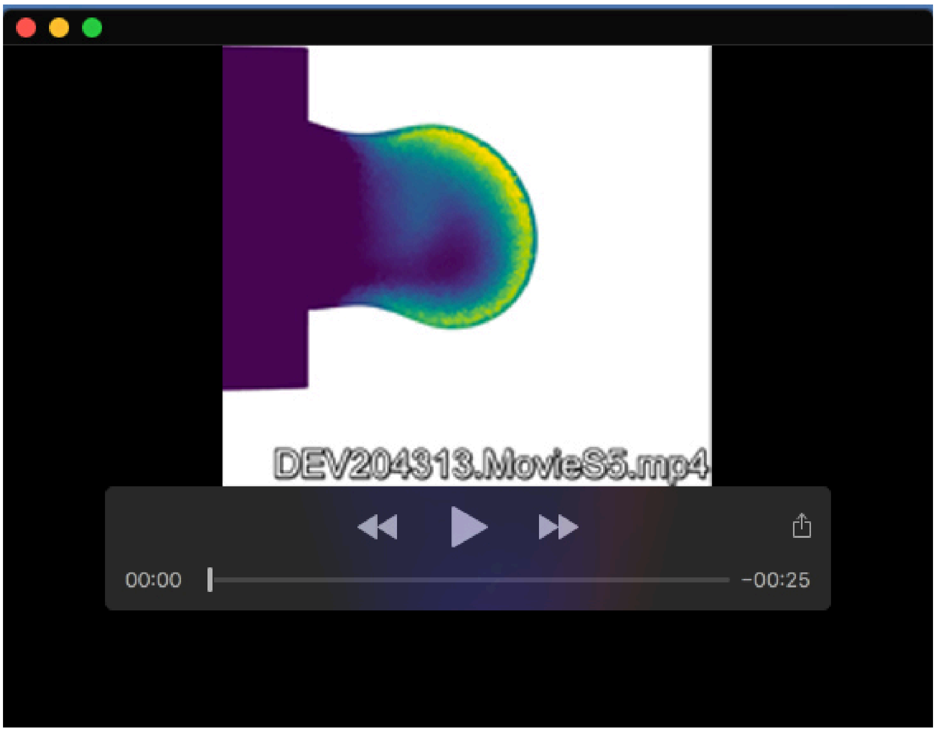

Movie 5. Interpolation of gene Dusp6 expression from developmental stage E10:20 to developmental stage E12:04. The video demonstrates the temporal progression with each frame spaced one developmental hour apart. A total of 20 digitized images were utilized to create this interpolation.

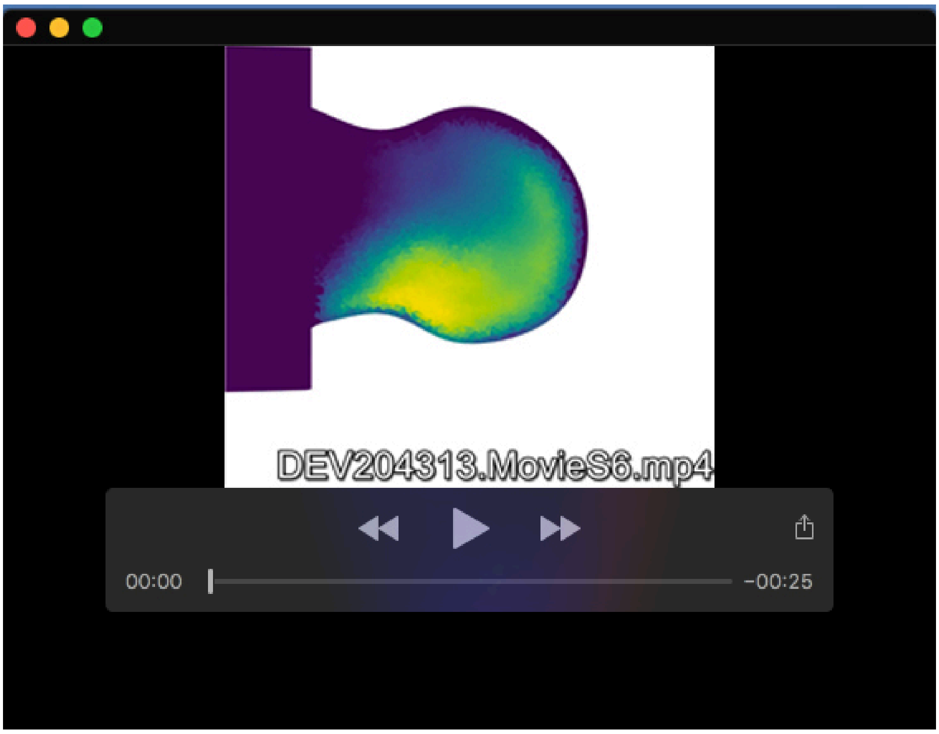

Movie 6. Interpolation of gene Hand2 expression from developmental stage E10:21 to developmental stage E12:00. The video demonstrates the temporal progression with each frame spaced one developmental hour apart. A total of 16 digitized images were utilized to create this interpolation.

Development • Supplementary information

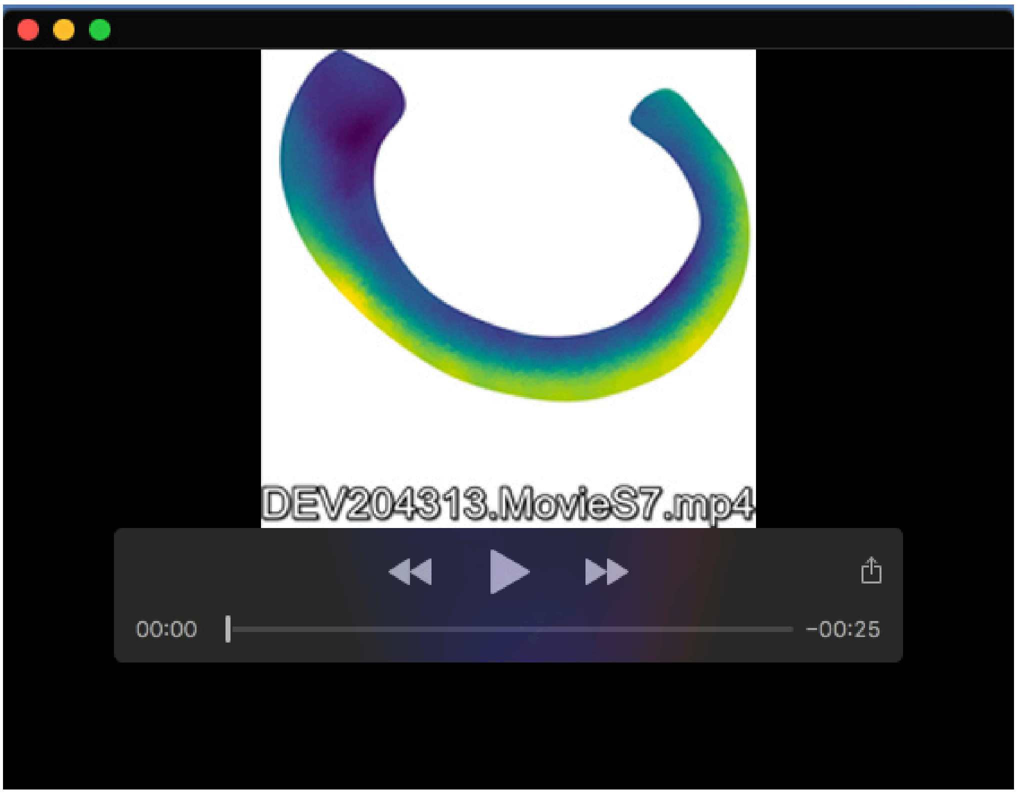

Movie 7. Interpolation of gene Hoxa7 expression on the neural tube. A total of 4 digitized images were utilized to create this interpolation.

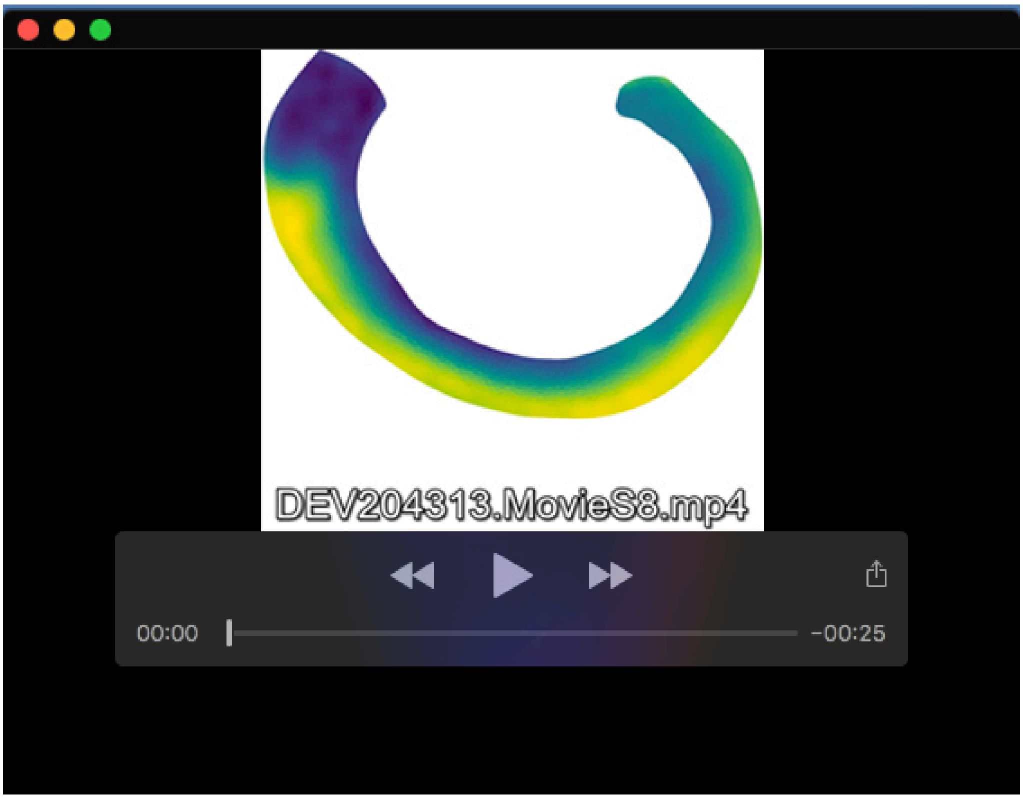

Movie 8. Interpolation of gene Hoxb6 expression on the neural tube. A total of 3 digitized images were utilized to create this interpolation.

Development • Supplementary information

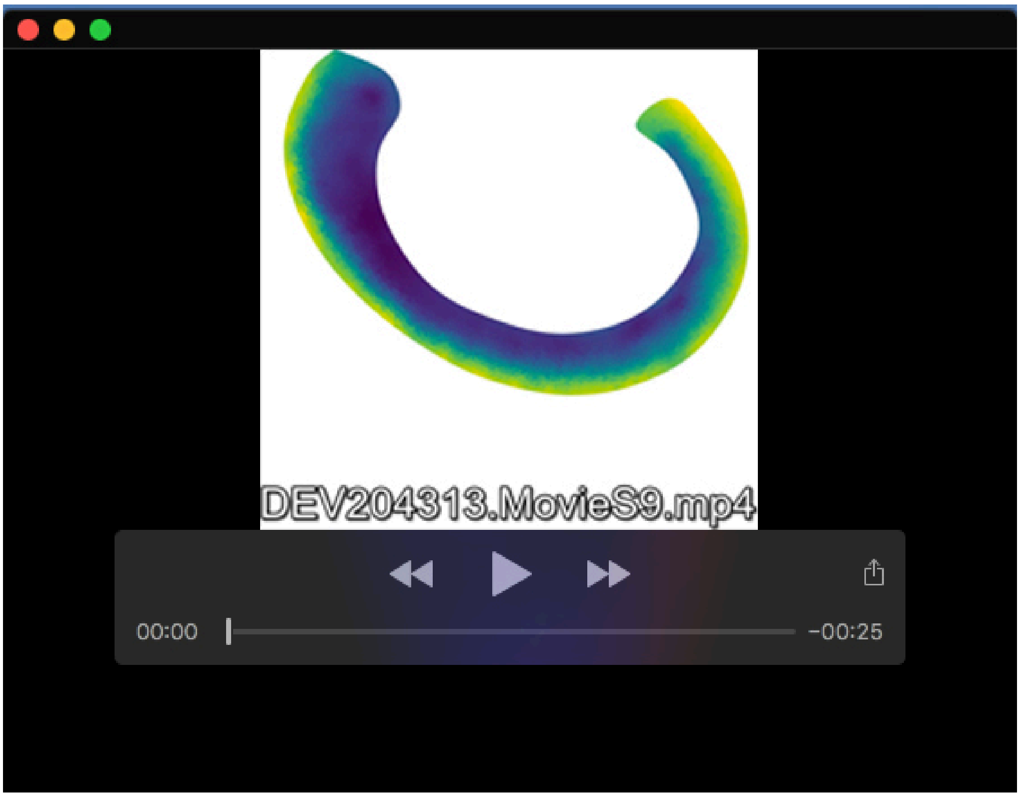

Movie 9. Interpolation of gene Fzd10 expression on the neural tube. A total of 3 digitized images were utilized to create this interpolation.

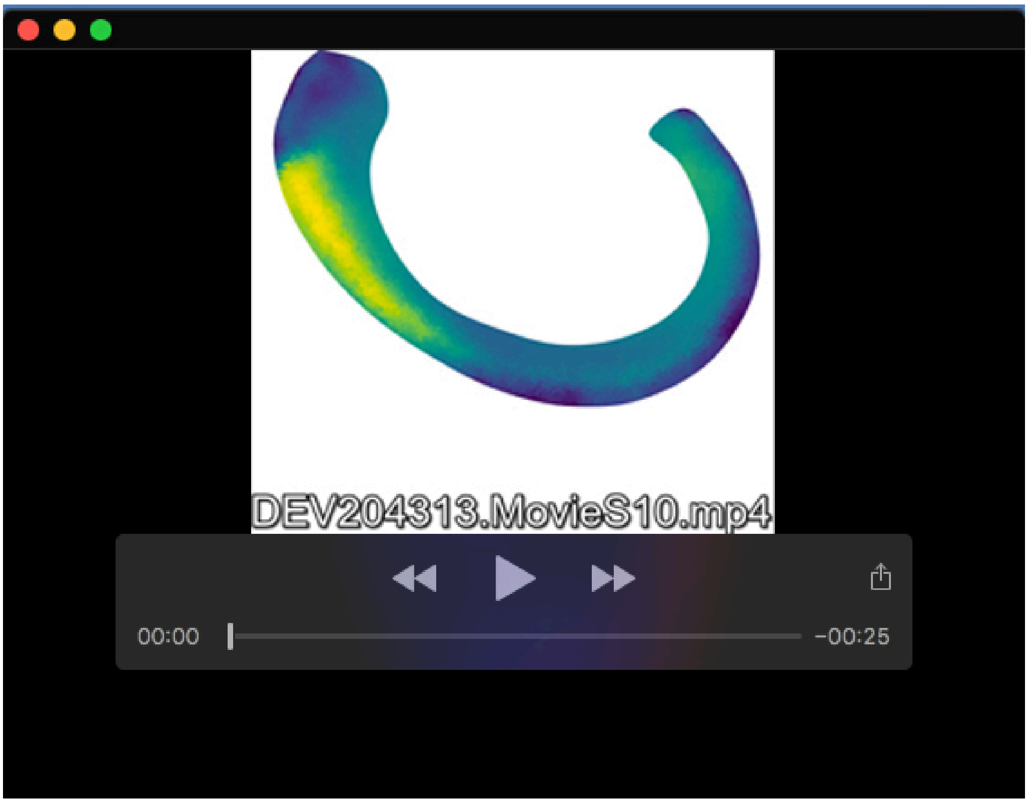

Movie 10. Interpolation of gene Raldh2 expression on the neural tube. A total of 3 digitized images were utilized to create this interpolation.
