# Supplementary material for: Spatio-temporal reconstruction of gene expression patterns in developing mice
Source: Development. 2025 Feb 21;152(4):DEV204313. doi: 10.1242/dev.204313 (PMC11883288; doi:10.1242/dev.204313)
Supplement: Supplementary file 1 [file develop-152-204313_review_history.pdf]

Spatio-temporal reconstruction of gene expression patterns in developing mice

Laura Aviñó-Esteban, Heura Cardona-Blaya and James Sharpe
DOI: 10.1242/dev.204313

Editor: Anna-Katerina Hadjantonakis

Review timeline
Original submission: 7 August 2024
Editorial decision: 22 September 2024
First revision received: 19 December 2024
Accepted: 16 January 2025

Original submission

First decision letter

MS ID#: dev.204313

MS TITLE: Spatio-temporal Reconstruction of Gene Expression Patterns in Developing Mice
AUTHORS: Laura Aviñó-Esteban and James Sharpe

I have now received all the referees' reports on the above manuscript, and have reached a decision. The referees' comments are appended below, or you can access them online: please go to:

As you will see, the referees express considerable interest in your work, but have some significant criticisms and recommend a substantial revision of your manuscript before we can consider publication. If you are able to revise the manuscript along the lines suggested, which may involve further experiments, I will be happy receive a revised version of the manuscript. Your revised paper will be re-reviewed by one or more of the original referees, and acceptance of your manuscript will depend on your addressing satisfactorily the reviewers' major concerns. Please also note that Development will normally permit only one round of major revision. If it would be helpful, you are welcome to contact us to discuss your revision in greater detail. Please send us a point-by-point response indicating your plans for addressing the referees’ comments, and we will look over this and provide further guidance.

Please attend to all of the reviewers' comments and ensure that you clearly highlight all changes made in the revised manuscript. Please avoid using 'Tracked changes' in Word files as these are lost in PDF conversion. I should be grateful if you would also provide a point-by-point response detailing how you have dealt with the points raised by the reviewers in the 'Response to Reviewers' box. If you do not agree with any of their criticisms or suggestions please explain clearly why this is so.

Comments from the Reviewers

Reviewer 1: SUMMARY OF THE ADVANCE MADE IN THIS PAPER AND ITS POTENTIAL SIGNIFICANCE TO THE FIELD

Understanding the dynamics controlling embryonic development is a major goal of developmental biology. Clearly, a major hurdle to this understanding is the inability of collecting longitudinal data on the same embryo. That is of course the case for many mammalian embryos that develop in utero. Thus, computational methods that allow reconstructing gene expression dynamics from fixed

© 2025. Published by The Company of Biologists under the terms of the Creative Commons Attribution License (https://creativecommons.org/licenses/by/4.0/).

datasets (e.g. in situs analysis of mRNA expression) are likely to be impactful. In this paper, the authors present a model for reconstructing gene expression dynamics in mouse limb. The method is exciting because it is both comprehensively addressing most of the challenges but also doing so using rather standard and well-established methods. Thus, I feel confident that the inferences presented in this paper are reasonable and not allucinations of complex over-fitting algorithms, as it can happen when using complex machine-learning or other multi-dimensional data analysis routines. Overall, I find that the methods used are reasonable, the limitations and challenges properly explained, and conclusions are properly drawn, so I am supportive of this paper.

SUGGESTIONS TO AUTHORS
The only suggestion I have stems from the fact that I was forced to look at the previous publication of the lab describing how the triangular mesh of the tissue is implemented. I think that a major goal of a Technique paper is to present the Technique in a way that facilitates as much as possible the usage by other researchers. I understand that it would not be appropriate to fully rewrite published results, but I would strongly urge the authors to give a short explanation of the method in their methods section and an intuitive explanation of how the mesh is derived in the main text. As an example, the authors provide a nice explanation of why they use splines rather than other fits (polynomial or piece-wise linear fits), even though that is clearly basic knowledge among computational scientists. That explanation will be helpful for most readers who might want to use their Technique. Similarly giving some background about the generation of the mesh would improve the paper.

Reviewer 2: SUMMARY OF THE ADVANCE MADE IN THIS PAPER AND ITS POTENTIAL SIGNIFICANCE TO THE FIELD

In this manuscript Esteban and Sharpe describe a new method for modeling dynamic patterns of gene expression based on static images and test them using several well-characterized genes in limb buds. Building on several previous methods from the Sharpe lab that provide accurate staging of limb buds, digitization of in situ images and morphomovie, they describe an approach for providing dynamic model of gene expression that is fitted to two-dimensional data using interpolation.

Strengths of the study include validation with several different gene expression datasets and a clear exploration of the limitations of the approach. This method will be useful to scientists interested in 2-D modeling of gene expression patterns, especially in the limb bud and potentially beyond. One of the limitations in the current study is that the approach is constrained to limb buds as opposed to being tested for broader utilization in other organs/developmental systems. A second limitation is that this approach in its current form approximates the limb bud as 2-D system even though many genes (such as Gremlin's expression in superficial mesoderm and exclusion from more central mesoderm) are more regional. While traditional in situ data relies on colorimetric readouts that limited acquisition of 3D information, this is no longer the case with fluorescent situ approaches, which are being rapidly adopted by the field. Taken together, this approach, while certainly useful, is currently limited in its broader usefulness to the field.

SUGGESTIONS TO AUTHORS

1. I appreciate the usefulness of the limb bud system where there are well-curated datasets and enough context for modeling. But it would also be interesting to know if this method can easily be applied to other developmental tissues where it would be much more broadly useful to the scientific community. Can this method easily be applied to other tissues beyond the limb bud? If so, please provide a proof-of-concept application.

2. As mentioned in the summary, this method seems somewhat dated given the issues with 2-D approximation of 3-D tissues and the ease of acquiring 3-D gene expression datasets with techniques like HCR. Is it possible to attempt a proof-of-concept application using Sox9 HCR images?

3. The code used for the approach should be provided on GitHub or another publicly accessible permanent repository.

© 2025. Published by The Company of Biologists under the terms of the Creative Commons Attribution License (https://creativecommons.org/licenses/by/4.0/).

4. The source data used for generating the models is from the Sharpe lab database. Could the specific data used for generating the datasets in the manuscript (especially Sox9) be provided as a supplemental dataset or at a public repository?

5. Page 2 line 17. FGF8 is described as having a 'morpho-gradient' and I'm wondering if they could clarify the meaning. As I understand it, Fgf8 mRNA has very sharp expression domains in the limb AER. Does this gradient behavior instead refer to FGF8-responsive genes?

6. Figure 3 legend wording. "The interpolation is [map] back to the morphomovie"

7. Figure 5. The artefact indicated by the arrowhead is not obvious to me. Can you explain (or zoom in on the feature)?

8. Page 16 line 177. Typo 'significant' instead of 'significantly'.

Reviewer 3: SUMMARY OF THE ADVANCE MADE IN THIS PAPER AND ITS POTENTIAL SIGNIFICANCE TO THE FIELD
The authors introduce a method of interpolating 2D in situ hybridisation results in space and time such that intermediate or data-deficient stages can be analysed in principle. This new method builds upon the group's other computational tools that permit visualisation of clonal expansion and morphogenesis of the limb bud and allows time-lapse-like visualisation of gene expression domains. The approach is valuable, arguably long overdue in the field, and likely accessible for many developmental biology labs that have access to an individual with a moderate degree of computational expertise. Overall, this will be a great addition to the field but I wonder whether the value of the method can be better explained on a few fronts to encourage greater interest and application by other labs.

SUGGESTIONS TO AUTHORS
The methodology is fairly well described for the general developmental biologist, including the somewhat challenging aspects concerning triangle splitting and backward tracking. Those sections could be somewhat better explained in part by bringing in a little more detail from the cited computational tools where triangle segmentation was introduced. The gene expression domains to which the approach is applied are useful examples given the mix of relatively simple and complex patterns.

The reason that smooth interpolation is useful in the first place is given in a general way that may seem vague to some investigators. If the authors have more specific applications in mind, such as possibly narrowing the parameter space in silico for hypotheses about pattern formation by morphogen gradients or their interactions, it could be useful to mention them to stimulate more excitement.

The method of validation of either the basic protocol or the boot strap approach can be better explained or justified. (It's not immediately obvious how one declares that a certain finding is an artefact as in Fig. 5E.) Alternatively, such a validation could be proposed for individual applications. For example, is there a gold standard-like in situ hybridisation outcome or repository data to which one could or should compare the outcome at known stages before embarking on interpolation?

In a similar vein, the ability of the method to deal with poor quality image data is very important, yet it is not entirely clear how a user should evaluate validity of the reconstruction outcome.

At the discretion of the authors, the discussion can include mention of how this approach can be extended to quantitative methods of in situ hybridisation or to spatial transcriptomics.

Also, discussion of the potential value of the approach to predict mis-patterned domains and skeletal patterns of mutants at stages for which data are not available due to lethality or embryo scarcity could be useful.

© 2025. Published by The Company of Biologists under the terms of the Creative Commons Attribution License (https://creativecommons.org/licenses/by/4.0/).

First revision

Author response to reviewers' comments

Reviewer #1:

SUMMARY OF THE ADVANCE MADE IN THIS PAPER AND ITS POTENTIAL SIGNIFICANCE TO THE FIELD

Understanding the dynamics controlling embryonic development is a major goal of developmental biology. Clearly, a major hurdle to this understanding is the inability of collecting longitudinal data on the same embryo. That is of course the case for many mammalian embryos that develop in utero. Thus, computational methods that allow reconstructing gene expression dynamics from fixed datasets (e.g. in situs analysis of mRNA expression) are likely to be impactful. In this paper, the authors present a model for reconstructing gene expression dynamics in mouse limb. The method is exciting because it is both comprehensively addressing most of the challenges but also doing so using rather standard and well-established methods. Thus, I feel confident that the inferences presented in this paper are reasonable and not hallucinations of complex over-fitting algorithms, as it can happen when using complex machine-learning or other multi-dimensional data analysis routines. Overall, I find that the methods used are reasonable, the limitations and challenges properly explained, and conclusions are properly drawn, so I am supportive of this paper.

Comment 1: The only suggestion I have stems from the fact that I was forced to look at the previous publication of the lab describing how the triangular mesh of the tissue is implemented. I think that a major goal of a Technique paper is to present the Technique in a way that facilitates as much as possible the usage by other researchers. I understand that it would not be appropriate to fully rewrite published results, but I would strongly urge the authors to give a short explanation of the method in their methods section and an intuitive explanation of how the mesh is derived in the main text. As an example, the authors provide a nice explanation of why they use splines rather than other fits (polynomial or piecewise linear fits), even though that is clearly basic knowledge among computational scientists. That explanation will be helpful for most readers who might want to use their Technique. Similarly giving some background about the generation of the mesh would improve the paper.

Response: Thank you for your very positive feedback on our manuscript. Regarding the specific comment, we have added a new section called "Morphomovie" to the Methods section and clarified how the meshes were generated. We believe this change helps improve the explanation and makes it easier to understand the process behind mesh generation, while still including the reference to the original parer, and the subsequent ones which used the same technique.

Reviewer #2:

SUMMARY OF THE ADVANCE MADE IN THIS PAPER AND ITS POTENTIAL SIGNIFICANCE TO THE FIELD

In this manuscript Esteban and Sharpe describe a new method for modeling dynamic patterns of gene expression based on static images and test them using several well-characterized genes in limb buds. Building on several previous methods from the Sharpe lab that provide accurate staging of limb buds, digitization of in situ images and morphomovie, they describe an approach for providing dynamic model of gene expression that is fitted to two-dimensional data using interpolation.
Strengths of the study include validation with several different gene expression datasets and a clear exploration of the limitations of the approach. This method will be useful to scientists interested in 2-D modeling of gene expression patterns, especially in the limb bud and potentially beyond. One of the limitations in the current study is that the approach is constrained to limb buds as opposed to being tested for broader utilization in other organs/developmental systems. A second limitation is that this approach in its current form approximates the limb bud as 2-D system even though many genes (such as Gremlin's expression in superficial mesoderm and exclusion from more central mesoderm) are more regional. While traditional in situ data relies on colorimetric

© 2025. Published by The Company of Biologists under the terms of the Creative Commons Attribution License (https://creativecommons.org/licenses/by/4.0/).

readouts that limited acquisition of 3D information, this is no longer the case with fluorescent situ approaches, which are being rapidly adopted by the field. Taken together, this approach, while certainly useful, is currently limited in its broader usefulness to the field.

- **Comment 1: I appreciate the usefulness of the limb bud system where there are well-curated datasets and enough context for modeling. But it would also be interesting to know if this method can easily be applied to other developmental tissues where it would be much more broadly useful to the scientific community. Can this method easily be applied to other tissues beyond the limb bud? If so, please provide a proof-of-concept application.**
  Response: This method can be applied to any arbitrary shape with relative ease, so we have decided to follow the reviewer´s request, doing a proof-of-concept by applying the method to the neural tube. To keep the focus of the manuscript on the main topic of the paper, we have added the new proof-of-concept work into the supplementary text, where we added a section describing the overall process of generating the neural tube morphomovie and testing the interpolation method with four different genes. We have included a figure as supplementary material and presented the results of the interpolation as Supplementary Videos 7-10. However, we are happy to move this content to the main text if the editor deems it more appropriate.
- **Comment 2: As mentioned in the summary, this method seems somewhat dated given the issues with 2-D approximation of 3-D tissues and the ease of acquiring 3-D gene expression datasets with techniques like HCR. Is it possible to attempt a proof-of-concept application using Sox9 HCR images?**
  Response: We agree that using up-to-date techniques, such as HCRs, can be beneficial, so we have decided to follow the reviewer's suggestion of adding some Sox9 HCR images into the process. As a result, in the revised manuscript we have now made clear that any source of 2D expression data can be used. We have created a new Supplementary Figure which explains how we can transform 3D fluorescence HCR data into 2D projection data. Moreover, we have now included data from three Sox9 HCR samples. This addition is explained in the Results section under "Intensity Mapping and Interpolation" and is also shown in Supplementary Figure 1. We have therefore recalculated the results shown in Figure 4 and 5 and supplementary Video 1 (which do not look visibly different).
- **Comment 3: The code used for the approach should be provided on GitHub or another publicly accessible permanent repository. The source data used for generating the models is from the Sharpe lab database. Could the specific data used for generating the datasets in the manuscript (especially Sox9) be provided as a supplemental dataset or at a public repository?**
  Response: In response to this suggestion, we have added a “Data Availability” section to the manuscript, providing the necessary information to access the data and code used in the study.
- **Comment 4: Page 2 line 17. FGF8 is described as having a 'morpho-gradient' and I'm wondering if they could clarify the meaning. As I understand it, Fgf8 mRNA has very sharp expression domains in the limb AER. Does this gradient behavior instead refer to FGF8-responsive genes?**
  Response: We appreciate the comment highlighting FGF8 is not a good example of the gradient nature of genes involved in morphogenesis - although the FGF8 ligand is distributed in a gradient, Fgf8 expression is not. In alignment with this observation, we have revised the manuscript to use WNT5a as the example gene, which is indeed expressed in a gradient
- **Comment 5: Figure 3 legend wording. "The interpolation is [map] back to the morphomovie".**
  Response: Thanks for noticing the mistake - we have corrected it now.
- **Comment 6: Figure 5. The artifact indicated by the arrowhead is not obvious to me. Can you explain (or zoom in on the feature)?**
  Response: To address the reviewer’s concern and clarify our use of the term "artifact", we have added 2 extra panels F and G to Figure 5, which provide a closer view of the feature with adjusted image exposure to better highlight it. Additionally, we have included a more detailed explanation in the figure legend. We believe these changes effectively demonstrate how the bootstrapping step distinguishes and removes both real

© 2025. Published by The Company of Biologists under the terms of the Creative Commons Attribution License (https://creativecommons.org/licenses/by/4.0/).

and artificial noise.

- **Comment 7: Page 16 line 177. Typo 'significant' instead of 'significantly'.**
  Response: Thank you for pointing that out. We have corrected the typo.

Reviewer #3:

SUMMARY OF THE ADVANCE MADE IN THIS PAPER AND ITS POTENTIAL SIGNIFICANCE TO THE FIELD
The authors introduce a method of interpolating 2D in situ hybridisation results in space and time such that intermediate or data-deficient stages can be analyzed in principle. This new method builds upon the group's other computational tools that permit visualization of clonal expansion and morphogenesis of the limb bud and allows time-lapse-like visualization of gene expression domains. The approach is valuable, arguably long overdue in the field, and likely accessible for many developmental biology labs that have access to an individual with a moderate degree of computational expertise. Overall, this will be a great addition to the field but I wonder whether the value of the method can be better explained on a few fronts to encourage greater interest and application by other labs.

- **Comment 1: The methodology is fairly well described for the general developmental biologist, including the somewhat challenging aspects concerning triangle splitting and backward tracking. Those sections could be somewhat better explained in part by bringing in a little more detail from the cited computational tools where triangle segmentation was introduced. The gene expression domains to which the approach is applied are useful examples given the mix of relatively simple and complex patterns.**
  Response: In response to the suggestion, we have added new text to the section titled “Tracking Tissue Movements” and also added a new section titled "Morphomovie" to the Methods section, where we have clarified the process of mesh generation. This addition aims to address the concern and improve the manuscript by making the mesh generation process more accessible and easier to understand without requiring reference to the original paper.
- **Comment 2: The reason that smooth interpolation is useful in the first place is given in a general way that may seem vague to some investigators. If the authors have more specific applications in mind, such as possibly narrowing the parameter space in silico for hypotheses about pattern formation by morphogen gradients or their interactions, it could be useful to mention them to stimulate more excitement.**
  Response: We appreciate the feedback on the usefulness of the method. In response to this concern, we have expanded the Discussion to include a more detailed explanation of the method’s potential applications. This revision incorporates additional applications not mentioned in the original manuscript and provides a more comprehensive discussion of the applications previously presented.
- **Comment 3 and Comment 4: The method of validation of either the basic protocol or the bootstrap approach can be better explained or justified. (It's not immediately obvious how one declares that a certain finding is an artifact as in Fig. 5E.) Alternatively, such a validation could be proposed for individual applications. For example, is there a gold standard-like in situ hybridisation outcome or repository data to which one could or should compare the outcome at known stages before embarking on interpolation? In a similar vein, the ability of the method to deal with poor quality image data is very important, yet it is not entirely clear how a user should evaluate validity of the reconstruction outcome..**
  Response: In general terms, the reviewer brings up an interesting and intrinsic “chicken-and-egg” problem in the analysis of gene expression patterns. Although metrics exist to compare 2 individual (non-spatial) expression levels from bulk tissue (eg. from RNA-Seq), to our knowledge there are no standardised metrics for comparing the spatial patterns of 2 genes (although the Sharpe lab has made contributions to this topic in previous papers (Uzkudun 2015, Martínez-Abadías 2016, Martínez-Abadías 2018). Our study introduces a method for quantitatively comparing two patterns, in our case, the reconstructed pattern and the gene expression data. It is outlined in the new section of Materials and Methods “Reconstruction accuracy”. Since the collected data can have varying intensity profiles due to different techniques or lighting conditions during image capture, we first

© 2025. Published by The Company of Biologists under the terms of the Creative Commons Attribution License (https://creativecommons.org/licenses/by/4.0/).

match the intensity profiles by applying a pairwise linear transformation to the reference image intensities to match them with the reconstructed shape intensities. This is done by minimizing the sum of squared residuals (SSD) between the gene expression data and the reconstruction. The minimum SSD is used as a metric for the accuracy of the reconstructed pattern in Figure 7d. However, the main challenge lies in defining whether “gold standard” data exist for a given gene. In general terms, for arbitrary genes, this does not appear to be the case. However, our analysis in Figure 7 is the closest we can reach: We digitise gene expression patterns for a series of time-points. Some of these time-points are then used to make trajectories, while the data from other time-points are left out. We then compared the predicted patterns with the real ones that were left out, to see how good the predictions were. The results of these comparisons are in the figure, and show that the predicted trajectories appear to be good. In parallel, we also acknowledge the challenge in interpreting what we mean by "artifact" in Figure 5. To clarify it, we have added panels F and G to Figure 5, which provide a zoomed-in view of the feature, along with a more detailed explanation in the figure legend. We believe these additions effectively demonstrate how the bootstrapping step distinguishes and removes both genuine and artificial noise.

- **Comment 5: At the discretion of the authors, the discussion can include mention of how this approach can be extended to quantitative methods of in situ hybridisation or to spatial transcriptomics.**
  Response: We agree that incorporating quantitative data into the input of our method is an excellent idea to improve the final results. In response to this suggestion, we have added a new paragraph (paragraph 4) in the Discussion section. In this paragraph, we explore potential extensions of the method to other data types and discuss how these extensions could enhance the accuracy and quality of the interpolation results.
- **Comment 6: Also, discussion of the potential value of the approach to predict mis-patterned domains and skeletal patterns of mutants at stages for which data are not available due to lethality or embryo scarcity could be useful.**
  Response: This is a great idea. We believe that this method could be used to perform interpolations of gene expression patterns in mutants, thereby improving the description of these patterns even with limited data points. However, it is important to note that this approach would only be applicable when the overall morphology of the limb remains relatively unchanged, as the interpolation is performed based on the wild-type morphomovie.

Second decision letter

MS ID#: dev.204313R1

MS TITLE: Spatio-temporal Reconstruction of Gene Expression Patterns in Developing Mice
AUTHORS: Laura Aviñó-Esteban, James Sharpe and Heura Cardona Blaya

I am happy to tell you that your manuscript has been accepted for publication in Development, pending our standard publication integrity checks.

Comments from the Reviewers:
Reviewer 2: This revised version has fully addressed my concerns from the first round of review and provides a nice addition to the field.

Reviewer 3: SUMMARY OF THE ADVANCE MADE IN THIS PAPER AND ITS POTENTIAL SIGNIFICANCE TO THE FIELD

The authors have fully addressed my questions. The paper is an important contribution. Best wishes.

© 2025. Published by The Company of Biologists under the terms of the Creative Commons Attribution License (https://creativecommons.org/licenses/by/4.0/).
